# Shape Deformation, Budding and Division of Giant Vesicles and Artificial Cells: A Review

**DOI:** 10.3390/life12060841

**Published:** 2022-06-06

**Authors:** Ylenia Miele, Gábor Holló, István Lagzi, Federico Rossi

**Affiliations:** 1 Department of Chemistry and Biology “A. Zambelli”, University of Salerno, Via Giovanni Paolo II 132, 84084 Fisciano, Italy; ymiele@unisa.it; 2 MTA-BME Condensed Matter Research Group, Budapest University of Technology and Economics, Muegyetem rkp. 3, 1111 Budapest, Hungary; hollo.gabor@edu.bme.hu; 3 Department of Physics, Institute of Physics, Budapest University of Technology and Economics, Muegyetem rkp. 3, 1111 Budapest, Hungary; 4 Department of Earth, Environmental and Physical Sciences—DEEP Sciences, University of Siena, Pian dei Mantellini 44, 53100 Siena, Italy

**Keywords:** giant vesicles, division, protocells, artificial cells, systems chemistry, budding, ADE theory

## Abstract

The understanding of the shape-change dynamics leading to the budding and division of artificial cells has gained much attention in the past few decades due to an increased interest in designing stimuli-responsive synthetic systems and minimal models of biological self-reproduction. In this respect, membranes and their composition play a fundamental role in many aspects related to the stability of the vesicles: permeability, elasticity, rigidity, tunability and response to external changes. In this review, we summarise recent experimental and theoretical work dealing with shape deformation and division of (giant) vesicles made of phospholipids and/or fatty acids membranes. Following a classic approach, we divide the strategies used to destabilise the membranes into two different types, physical (osmotic stress, temperature and light) and chemical (addition of amphiphiles, the addition of reactive molecules and pH changes) even though they often act in synergy when leading to a complete division process. Finally, we review the most important theoretical methods employed to describe the equilibrium shapes of giant vesicles and how they provide ways to explain and control the morphological changes leading from one equilibrium structure to another.

## 1. Introduction

Cells are the most amazing chemical laboratories developed over billions of years of natural evolution. Even the simplest biological cells are able to maintain and organise thousands of parallel processes structured in chemical networks and accomplish complex tasks essential for life, such as metabolism, maintaining homeostasis, growth, self-division, self-replication, and adapting and responding to environmental stimuli. In order to master processes of increasing complexity, chemists are increasingly taking inspiration from nature to shift from a reductionist towards a systemic approach for designing new research protocols and achieving technological and scientific goals. Therefore, fast-growing disciplines, such as synthetic biology and systems chemistry, are creating and engineering artificial and synthetic systems able to reproduce life functions based on bottom-up approaches [1,2,3,4]. This new view showed to be very successful for the design of nano- and micro-objects (particles, micelles, vesicles, etc.) and their applications (e.g., smart materials, targeted drug delivery, biocompatible devices, etc.) [5,6,7,8]. Moreover, a systems chemistry approach to cell imitation is thought to be a valuable contribution to understanding fundamental open questions in the origin of life studies [2,9].

A few general hallmarks of cells [10,11,12,13,14,15,16] have been shortlisted as essential prerequisites that a synthetic or artificial system needs to mimic, i.e., being (i) far-from-equilibrium and dissipative; (ii) confined and compartmentalised, yet able to communicate and exchange matter and information with the environment and (iii) governed by nonlinear mechanisms and kinetics. Within this framework, systems chemists generally adopt a bottom-up approach to design chemical systems (or protocells) able to self-assemble in highly ordered supramolecular structures and/or to produce self-organised behaviours that accomplish complex functions without replicating the complex biological environment typical of the living cells. In this way, distinctive processes that characterise modern cells were successfully reproduced: these include, but are not limited to, collective behaviour and communication [17,18,19,20,21,22,23], energy harvesting [24,25,26] and metabolism [27,28]. Another common natural mechanism that has been extensively studied and mimicked by using synthetic systems is self-reproduction or division, which is the subject of the next sections of this paper.

Giant vesicles (GVs, or Giant Unilamellar Vesicles, GUVs), i.e., water-in-water compartments defined by an amphiphilic bilayer made of phospholipids (liposomes), fatty acids, synthetic polymers (polymersomes) or their combinations (hybrid vesicles), are becoming the most used model for studying artificial cells [29]. Their popularity rapidly increased over the last 20 years, mainly because they allow for real-time observation of the cellular dynamics through simple optical microscopes. In fact, depending on the preparation method, GV size is typically within the range 1–100 μm, with a variable degree of polydispersity, which is high in the case of bulk (electroformation, natural swelling and gentle hydration) and inverse phase methods, or it is low when GVs are obtained by means of microfluidic techniques. A homogeneous population can, nevertheless, be selected through post-synthetic procedures, such as extrusion or chromatographic separation [30,31,32,33,34]. A proper design of the composition of a GV membrane is important for studying the processes of shape deformation, which eventually leads to division [35,36], and this aspect contributed to boosting the research of more sophisticated stimuli-responsive membranes, which is having a great impact on technological applications, especially those based on lipid chemistry [37,38,39]. In addition to GVs, unilamellar small vesicles (SUVs, *d*∼ 20–100 nm), large (LUVs, *d*∼ 100 nm–1 μm) and multilamellar large vesicles (MLVs) are widely employed as models for studying the shape deformations of cells.

In general, division or self-division is a statistical process by which cells, vesicles or micelles deform until a new structure is formed, not necessarily with identical composition or size. In contrast, when some of the parent traits are inherited by the offspring, the term self-reproduction is used. In addition, in the case of self-reproduction, the daughter units are intended as completely separated, while after budding processes, the daughter units are still connected by a narrow neck. It is also necessary to distinguish self-reproduction from self-replication, which involves a copy of the molecule carrier of genetic information such as DNA or RNA [40,41]. Biological cells self-reproduce by a complex mechanism, which involves a growth stage followed by the membrane fission, to produce more copies of the original cell and to transmit genetic material and other biomolecules necessary for the survival to the next cell generation. The division mechanisms, in particular, can be classified into two main categories depending on the energy requirement [36,42]: passive division is the result of the spontaneous reorganisation of the membrane-forming molecules while reaching a more favourable thermodynamic status; active division occurs through direct consumption of cellular energy as, for example, the membrane constriction operated by some proteins. Active division mechanisms were successfully reconstituted in the membrane of synthetic GVs by the group of P. Schwille [36,43,44,45,46].

However, in many attempts to mimic self-division in artificial cells, the most general strategy is to deform the membrane by influencing its elastic energy (see Section 4 for more details) [47,48,49,50,51,52,53,54,55]. Fission generally occurs when the volume of the vesicles decreases and, concurrently, the inner surface area of the inner bilayer leaflet decreases with respect to the outer one. In this paper, we review a few different approaches that have been proposed to induce synthetic GVs to bud and eventually divide, with an emphasis on the more recent advancements in the field. As in previous reviews [56,57], we distinguish between physical (osmotic stress, temperature and light, Section 2) and chemical (addition of amphiphiles, reactive molecules and pH changes, Section 3) triggers that have been employed to bring the GUVs membrane out of the equilibrium, even though a strict differentiation between the two is rather difficult and in some cases they overlap and fade one into the other. We also briefly review the theoretical approaches developed to model and describe the shape changes of vesicles, often derived from the minimisation of the membrane elastic energy.

## 2. Physical Stimuli

Temperature gradient and osmotic stress are the most investigated physical stimuli used to destabilise single-phase or phase-separated giant phospholipid vesicles. In general, they are the simplest experimental means to change the area to volume ratio for altering the elastic energy of the membrane and inducing some change in the equilibrium shape of the vesicles, possibly leading to budding or division. Early attempts to experimentally control the GUVs shape by external stimuli were reported by Sackmann et al. [58] for a DMPC membrane undergoing a heating cycle. This report was among the first experimental confirmation that equilibrium shapes of a vesicle can be described in terms of the area to volume ratio [48]. The temperature mainly influences the surface area of the vesicles through the molecular organisation of the amphiphilic molecules. In the case of phospholipids, for example, at the main chain transition temperature (order-disorder transition or melting, *T*${}_{m}$), the acyl chains pass from an ordered gel phase, in which the chains are fully extended and closely packed, to a disordered liquid crystalline phase, where the chains are randomly oriented and fluid. In contrast, the osmotic stress causes a variation in the volume of the compartments, depending on whether the vesicle is surrounded by a hypotonic (volume increase) or a hypertonic solution (volume decrease). Osmolarity changes can be triggered by water evaporation, dilution processes, the addition of non-permeable molecules to the outer solutions or more elegant methods such as the enzymatic decomposition of sucrose [47,48,49,50,51,52,53,54,59,60]. Recently [61], in addition to the effect on the equilibrium shape, the osmotic deflation of giant vesicles was found to control the activity of biochemical reaction networks inside their lumen, thus providing further insight into how simple mechanisms could control complex functions in protocells.

**Figure 1:**
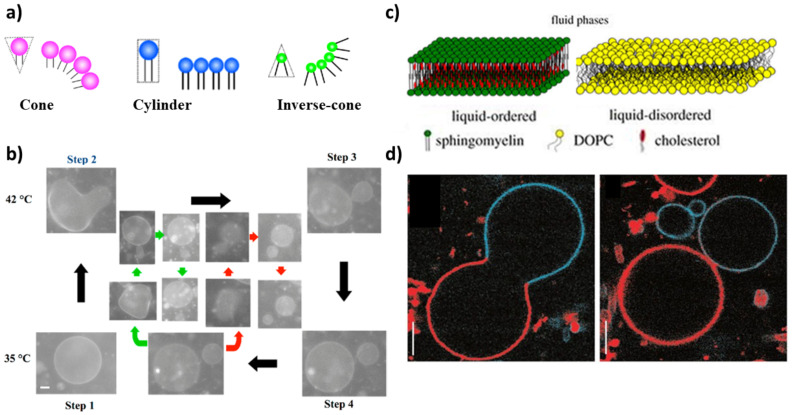
(**a**) Schematic representations of cone-, cylinder- and inverse-cone-shaped lipids. Reproduced from [35]. (**b**) Self-reproduction cycle for binary vesicles composed of DLPE/DPPC = 2/8. The green and red arrows show the budding of the second daughter vesicle and the granddaughter vesicle, respectively. Scale bar 5 μm. Reproduced from [35]. (**c**) Lipid bilayer in liquid-ordered phase (Lo) (composed of SM and cholesterol) and liquid-disordered phase Ld (composed of DOPC). Reprinted with permission from Ref. [66]. 2018, Royal Society of Chemistry. (**d**) Budding (left, *T* = 30 ${}^{\circ }$C) and complete budding (right, *T* = 35 ${}^{\circ }$C) of phase-separated vesicles made of sphingomyelin, DOPC and cholesterol. Scale bar 5 μm. Reprinted with permission from Ref. [65]. 2003, Springer Nature.

A further step towards the mimicking of a real cell membrane is to employ a mixed phospholipid membrane to constitute GVs. Lipids with different physical properties allow for the formation of domains that improve the control over the shape deformation and help to select more precisely the budding and/or division region. The proper choice of the shape of the membrane-forming amphiphiles can thus help in driving the division process of a mixed GUV following a temperature variation. From a geometrical point of view, in fact, the shape of amphiphilic molecules can be classified into three types: cone, cylinder and inverse cone (Figure 1a). Inverse cone-shaped lipids are preferentially distributed in the inner leaflet of a spherical vesicle, cone-shaped lipids preferentially go in the outer leaflet, while for cylinder-shaped lipids the symmetry of the bilayer does not dictate any geometrical preference [35,62]. A cyclic self-reproduction through a temperature change was observed in a mixed vesicle made of cylinder-shaped lipids with a high melting temperature (1,2-dipalmitoyl-*sn*-glycero-3-phosphocholine: DPPC, *T*${}_{m}$ = 41 ${}^{\circ }$C) and inverse-cone-shaped lipids with a low melting temperature (1,2-dilauroyl-*sn*-glycero-3-phosphoethanolamine: DLPE, *T*${}_{m}$ = 29 ${}^{\circ }$C) [63]. By heating the vesicles above the *T*${}_{m}$ of DPPC, the cross-sectional area of DPPC increases while the area of DLPE remains constant. The binary GUV deforms to a budded limiting shape, and after the rupture of the neck, two vesicles are formed. After cooling to the initial temperature, daughter vesicles recover the spherical shape; in the following cycle, the division process is repeated in both mother and daughter vesicles yielding several generations of vesicles (Figure 1b). The main factors that affect the process of self-reproduction are: (i) the distribution of the inverted-cone component within the membrane (DLPE molecules are preferentially distributed in the inner leaflet), (ii) the change of cross-section for DPPC [35,63]. The importance of the different geometry of lipids in binary vesicles DPPC/DLPE and the change of area has been theoretically demonstrated through the area difference elasticity theory (ADE) [54] and molecular dynamics simulations [64].

Complete budding was observed in ternary vesicles made of 1,2-dioleoyl-*sn*-glycero-3-phosphocholine (DOPC), sphingomyelin and cholesterol, though the process was not recursive (the composition changes after the deformation) [65]. Initially, the three components coexist in one phase (*T* > $T_{m}$); when demixing occurs after a temperature decrease, sphingomyelin and cholesterol go in the ordered phase, L${}_{o}$, while DOPC prefers a disordered phase, L${}_{d}$ (Figure 1c). The presence of domains with a different lipid composition results in a line tension at the domain boundary. The membrane deformation through budding processes depends on the competition between the bending energy and the line energy of the membrane. When the bending energy is dominant, the membrane prefers a flat geometry; when the line energy governs the system, the vesicle forms a bud to decrease the edge length of the domain, and then, the bud domain forms two vesicles (complete budding), where the line energy disappears [35,65] (Figure 1d).

**Figure 2:**
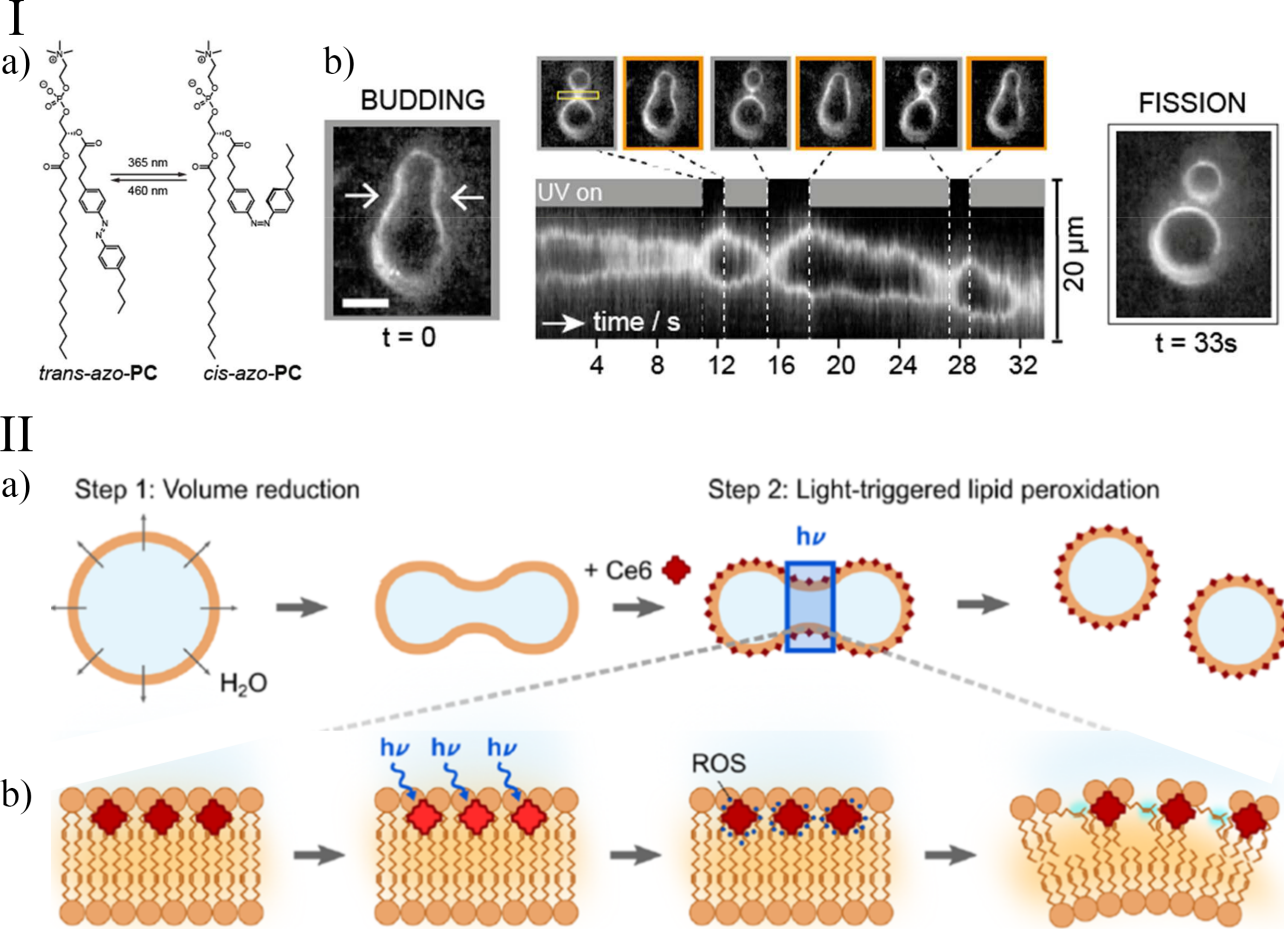
Light-triggered division of GUVs. Panel I: (**a**) An example of an azobenzene-containing phosphatidylcholine (*azo*PC) that can be isomerised between its cis- and trans-configurations; (**b**) budding transition of an *azo*PC vesicle following the illumination with either 365 or 460 nm light. The space-time plot demonstrates the reversibility of the process. After 33 s, vesicle fission is initiated by intense white light illumination. Adapted with permission from Ref. [67]. 2017, American Chemical Society. Panel II: (**a**) In the first step, GUVs deform after the addition of a higher osmolarity sucrose solution. In the second step, illumination leads to local lipid peroxidation of the outer membrane leaflet in the presence of the photosensitiser Ce6; (**b**) mechanism of Ce6-mediated lipid peroxidation. Illumination at the 405 nm wavelength triggers the generation of reactive oxygen species (ROS) in close proximity to the lipid tails. The ROS causes the peroxidation of the lipids in the outer leaflet and hence an asymmetric area increase. Reprinted with permission from Ref. [73]. 2021, American Chemical Society.

Light is another physical stimulus often used to trigger budding or self-division in GUVs. Recently, Pernpeintner et al. [67] achieved an impressive shape control over GUVs made of azobenzene-based phospholipids (*azo*PC [68]) through a light-induced photoswitchable cis-trans isomerisation of the membrane-forming molecules. *azo*PCs are azobenzene-derivatised phospholipids with a phosphatidylcholine head-group and one or both acyl chains containing the photoswitchable molecule. The trans form of the phenyl groups (with respect to the N=N double bond) is thermodynamically more stable in the presence of white light, and it confers to the amphiphilic molecule a linear shape that favours the membrane self-assembly. When irradiated with UV light (λ=365 nm), the trans to cis switch takes place and the acyl chain containing the azobenzene group assumes an L-shaped form that destabilises the membrane and affects its packing parameters and the bending energy. The azobenzene photoswitch is reversible, and once illuminated with a white or blue (λ=460 nm) light, the amphiphilic molecule turns back to the cis form and the membrane recovers the original packing and bending properties (Figure 2 Panel I: a). The reversibility of the isomer transition is reflected in a fine control of the shape of vesicles entirely made of *azo*PCs, which can be driven from a spherical form to a budded shape and finally to a division (UV illumination, trans to cis transition), but the process can be inverted to fuse back the two daughter vesicles and reconstitute the original GUV (Figure 2 Panel I: b). Interestingly, the intensity of the illumination can be used to control the kinetics of the photoswitch to obtain more dynamical behaviours of the GUVs, for example, invaginations, pearling and bursting. A similar behaviour, though with a lesser control over the membrane dynamics, has also been exerted when amphipathic azobenzene molecules were inserted in phospholipid-based GUVs [69,70,71,72].

Another strategy for light-controlling the membrane dynamics in GUVs was devised by Heuvingh and Bonneau [74] and recently reprised by Dreher et al. [73]. Instead of modifying the shape of a membrane-forming molecule, they inserted a photosensitiser (Chlorin e6, Ce6) in the inner or the outer leaflet of phase-separated GUVs, which, upon irradiation, induces the peroxidation of the lipids. Initially, GUVs are osmotically deflated to provide an excess membrane area sufficient for division. In a second step, a few seconds of illumination lead to local lipid peroxidation of the outer membrane leaflet, which increases the spontaneous curvature enabling neck fission (Figure 2). Under iso-osmotic conditions, in the presence of Ce6, a small bud is formed upon 405 nm laser illumination due to a small increase in the membrane area and spontaneous curvature; however, no division into equally sized compartments occurs.

In another approach, light was used to change the osmolarity of the solution instead of directly influencing the membrane of the vesicles. Phase separated vesicles that confine the non-fluorescent molecule bis-(5-carboxymethoxy-2-nitrobenzyl)-ether (CMNB)-fluorescein undergo division upon illumination at 405 nm [60]. At this wavelength, the CMNB-fluorescein splits into three components, two CMNB molecules and fluorescein, thus increasing the osmolarity in the lumen of the vesicle leading to the GUV division. In both the experiments with Ce6 and CMNB-fluorescein, the GUV deforms to minimise the energy associated with the line tension, a change of osmolarity leads to an increase in the surface-to-volume ratio and the contraction at the phase boundary with the formation of two daughter units.

Recently, a new kind of physical stimulus has been reported to induce shape changes from a spherical vesicle to twin vesicles connected by a tether when self-propelled Janus colloids are encapsulated in a DOPC giant vesicle. In this case, the deformations are caused by hydrodynamic active forces, which are locally induced on the membrane by the swimming particles [75].

## 3. Chemical Stimuli

A wide variety of chemical stimuli can promote the budding and division of vesicles: (i) addition of amphiphilic molecules into the surrounding solution, (ii) chemical reactions in the water pool or in the bilayer, (iii) change of pH or a combination of multiple stimuli. In most of these approaches, fatty acids and phospholipid vesicles have been adopted as artificial models of cells. In fact, fatty acid vesicles are considered, to date, the most plausible prebiotic compartments (monocarboxylic acids are simpler molecules compared to phospholipids; they were isolated from the Murchison meteorite and different reaction pathways have been proposed for their synthesis in abiotic conditions) [40,76,77,78]. Similarly to the physical stimuli strategy, the use of chemical triggers seeks to generate an imbalance between the surface area and the volume of the GVs population.

The strategy of adding amphiphilic molecules to preformed aggregates was first reported by the group of P.L. Luisi for an autopoietic system of fatty acids reverse micelles [79,80,81,82]. Successively, due to the massive development of experimental techniques which allow for monitoring the real-time dynamics of vesicular shape deformation, reverse micelles were progressively replaced with SUVs, LUVs, MLVs and GVs [4]. The encapsulation of oleate molecules through the addition of oleate micelles to a solution of pre-existing oleate/oleic vesicles at buffered pH [83] and pre-existing phospholipid vesicles (POPC:1-palmitoyl-2-oleoyl-*sn*-glycero-3-phosphocholine) [84] was found to lead to growth and division. More recently, the group of J.W. Szostak found that the division dynamics of oleate vesicles fed by micelles can be further catalysed by the presence of clay particles [85,86] and can undergo multiple growth and division cycles, which redistribute the encapsulated RNA within the daughter vesicles [87].

**Figure 3:**
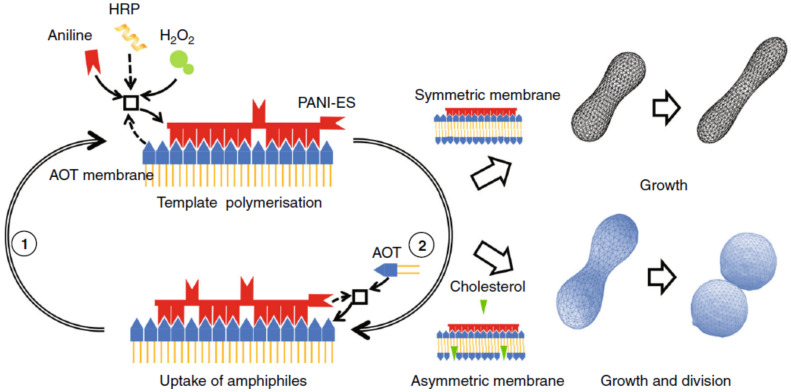
General scheme of the system AOT and PANI-ES. The polymerisation of aniline occurs on the surface of AOT vesicles. In symmetric membranes, the addition of AOT micelles leads to the deformation and growth of the vesicles; in asymmetric membranees, growth and division into two daughter vesicles are observed. Reproduced from [88].

Vesicles composed of AOT (sodium bis-(2-ethylhexyl) sulfosuccinate) coupled with an enzymatic polymerisation that occurs on the lipid surface undergo deformations after the addition of AOT micelles [88]. The enzymatic reaction is the horseradish peroxidase catalyzed polymerisation of aniline with hydrogen peroxide as oxidants, which gives polyaniline in the emeraldine form ([C${}_{6}$H${}_{4}$NH]${}_{2}$[C${}_{6}$H${}_{4}$N]${}_{2}$)${}_{n}$). In symmetric bilayers, the addition of AOT micelles induces the growth of the vesicles; if the bilayer is asymmetric, for example, with the introduction of cholesterol, which induces a negative spontaneous curvature, vesicles not only grow but self-reproduce (Figure 3).

A further way to change the number of amphiphilic molecules involves the synthesis in situ starting from proper precursors. Vesicles made of caprylic acid or oleic acid in water undergo autopoietic self-reproduction after the hydrolysis of caprylic or oleic anhydride [89]. The anhydride is insoluble in water, so it is introduced as oil. In the absence of vesicles or micelles, the reaction is extremely low, and the presence of vesicles catalyzes the hydrolysis of the anhydride, which leads to an increase in the number of surfactant molecules and consequently to the growth of the vesicles in size and number. The process of self-reproduction mediated by the hydrolysis of the oleic anhydride has been coupled with (i) the polymerisation of ADP to polyA catalyzed by polynucleotide phosphorylase [90]; (ii) the replication of an RNA template catalyzed by Qβ replicase [91]. Both these supramolecular systems can be seen as primitive models of a minimal cell. Four enzymes for the production of phosphatidylcholine (PC) were also encapsulated in phosphatydilcholine liposomes: *sn*-glycerol-3-phosphate acyltransferase, l-acyl-*sn*-glycerol-3-phosphate acyltransferase, phosphatidate phosphatase, and cytidinediphosphocholine phosphocholinetransferase. The incorporation of the newly PC synthesised molecules in the layers of the host liposomes results in the growth and division of the liposomes [92]. Moreover, phospholipids can be synthesised through acyl chain elongation via copper (I)- catalyzed azide-alkyne [3+2] cycloaddition reaction [93].

Similarly, in-situ phospholipids synthesis was obtained by using synthetic, self-reproducing catalysts capable of perpetuating phospholipid bilayer formation through the cycloaddition reaction [94]. The autocatalyst was a Cu complex of tris-(lauryl triazole)amine that was able to drive the lipid synthesis through the continuous formation of triazole phospholipids that favoured the growth of the vesicle and a stimuli-responsive behaviour. Coupled with pH changes, Cu-catalyzed alkyne-azide cycloaddition has also been investigated for dissipative self-assembly and self-reproduction of protocells [95]. Under hydrolysing conditions, the autocatalytic system is destroyed and the pH of the surrounding environment selects the final aggregation state of the protocells, which can self-reproduce in the form of vesicles or self-assembly into micelles.

**Figure 4:**
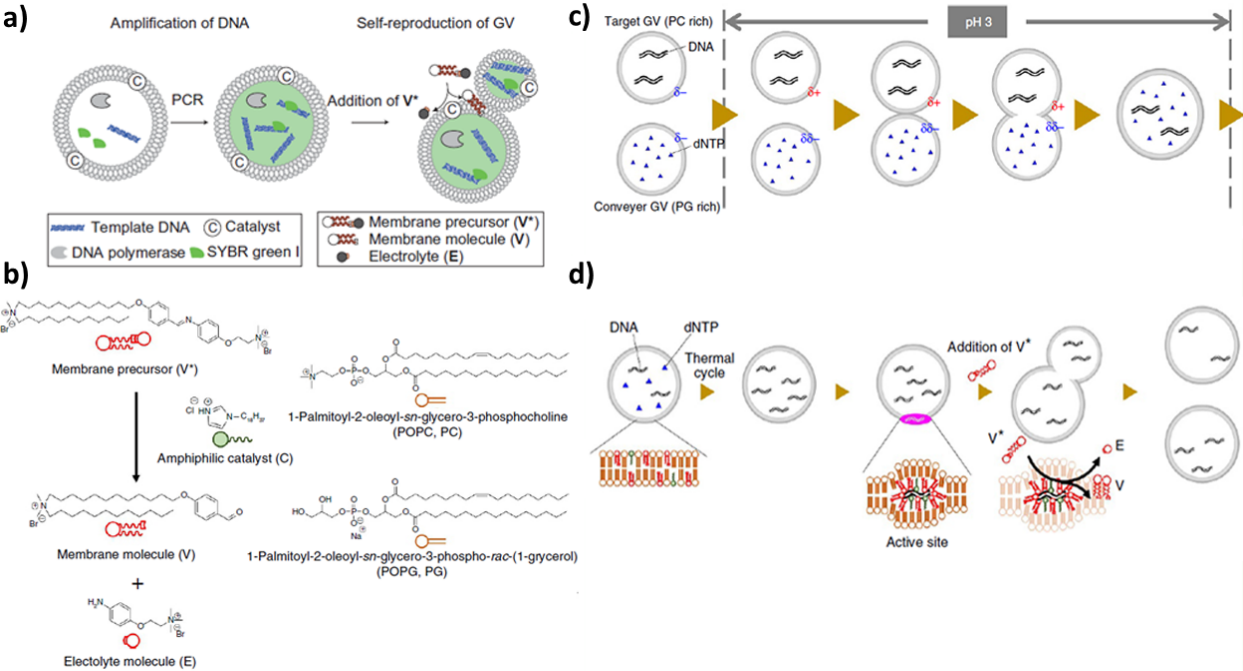
(**a**) Amplification of DNA within a GUV. Vesicles contain PCR reagents and a fluorescent probe: template DNA, primers, fluorescent tag SYBR Green I, deoxynucleoside triphosphates, DNA polymerase and Mg${}^{2+}$. Reprinted with permission from Ref. [96]. 2011, Springer Nature. (**b**) Chemical structures of membrane molecule V, amphiphile catalyst C, membrane precursor V* and electrolyte E. Reproduced from [97]. (**c**) Adhesion and fusion between a target GUV and a conveyer GUV. The surface charge of the target GUV changes to cationic due to the protonation of the POPC as well as the increase in the cationic membrane lipid V from its precursor. These two types of GUVs fuse, and the transport of dNTP from the conveyer GV to the target GUV proceeds. Reproduced from [97]. (**d**) Production of cationic membrane lipid V from its precursor V* after the transport mechanism depicted in (**c**). Reproduced from [97].

Self-reproduction of giant vesicles mediated by the synthesis of in situ amphiphilic molecules has been combined with self-replication of DNA [96]. Mixed vesicles composed of a zwitterionic phospholipid (POPC), a positive lipid (V), a negative phospholipid (POPG: 1-palmitoyl-2-oleoyl-*sn*-glycero-3-phosphoglycerol) and a catalyst C were prepared by a film swelling method in an optimised ratio: [POPC:POPG:V:C] = 6:2:2:1. Vesicles contained template DNA, primers, fluorescent tag SYBR Green I, deoxynucleoside triphosphates, DNA polymerase and Mg${}^{2+}$. When a bolaamphiphilic V* membrane precursor was added (boloamphiphilic means that the amphiphilic molecule has hydrophilic groups at both ends of the chain), V* was attracted from the negative DNA and C catalyzed the conversion of V* in V (Figure 4a and chemical structures in Figure 4b). Without thermal cycles, the GUVs rarely divided in two hours; applying thermal cycles but without polymerase, few vesicles divided. Therefore, DNA is essential for the growth and division of these GUVs. A more sophisticated transport system was developed by the same research group in 2015 [97]. A target GUV containing all of the reagents needed for DNA replication, except for deoxyribonucleotide triphosphates (dNTPs), adhered and fused at a pH of 3 with a ‘conveyer’ GUV filled with dNTPs (Figure 4c). After neutralisation, the vesicles were subjected to thermal cycles (Figure 4d). These two systems are good models for the Origin of Life research field: the self-reproduction process occurs without specific proteins in contrast with modern cells (for example, even simple organisms such as *Escherichia coli* express a special protein, FtsZ, that they arrange along the circumference of the cell for compression [98]), the stimuli applied (temperature and pH) could have been present in prebiotic environments such as hydrothermal vents.

Thus far, we have seen pH as a stimulus to generate adhesion of the vesicles and the transport of reactants. Can pH alone induce self-reproduction of vesicles?

**Figure 5:**
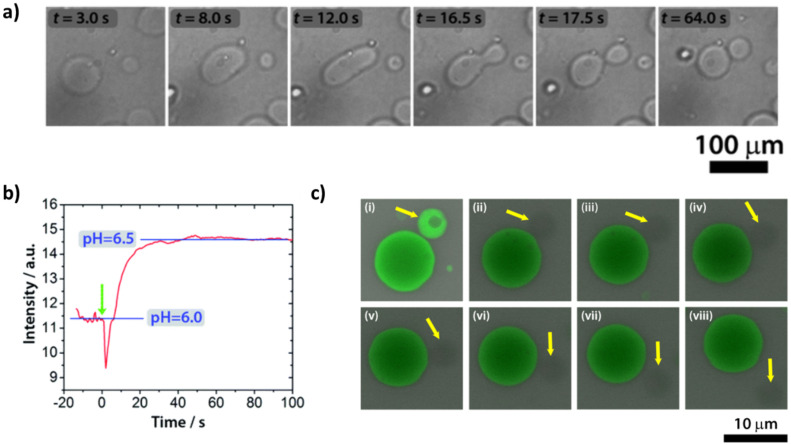
(**a**) Self−division of a GUV triggered by the urea–urease reaction. Transformation of a GUV from a spherical shape through prolate and pear shapes into two daughter vesicles. (**b**) Change of the fluorescence intensity of pyranine vs. time inside the GUVs. (**c**) FRAP (fluorescence recovery after photobleaching) experiment using fluorescein sodium salt as a fluorescent probe. One of the two daughter vesicles (indicated by a yellow arrow) was irradiated with a laser pulse after the division process. The lack of fluorescence recovery is proof of the effective separation between the two daughter vesicles. The time between consecutive snapshots is 30 s. (**a**,**b**) reproduced from [102], (**c**) reproduced from the Supplementary Information of [102].

Fatty acid vesicles composed of decanoic acid/decanoate (DA) vesicles show a series of shape deformations, i.e., prolate–oblate–stomatocyte–sphere, after a microinjection of a NaOH solution [99]. A slight deformation of the membrane was detected in vesicles of brain L-α-phosphatidylserine (PS) and egg yolk L-α-phosphatidylcholine (PC) [100]. Interesting structures formed after acid transport in vesicles of egg yolk L-α-phosphatidylcholine (PC), egg L-α-phosphatidylethanolamine (PE) and heart bovine 1,3-bis(*sn*-3-phosphatidyl)-*sn*-glycerol diphosphatidylglycerol (CL) in a ratio PC:PE:CL = 60:30:10 [101]. Membrane invagination and ‘‘cristae-like’’ structures such as the ones of mitochondria were monitored over time. A series of control experiments proved that CL is essential in the formation of cristae (these structures were not observed with other lipids) and are not a simple consequence of osmotic shocks (buffer or salt solutions did not give cristae). These results obtained with a minimal cell model shed light on the role of cardiolipin (CL) and pH gradients in the morphology and dynamics of mitochondria. All these approaches, based on a variation of pH that affects the outer leaflet of the bilayer, give shape changes but not self-reproduction. By changing the point of view, i.e., with a pH variation that affects the inner leaflet, our research group was able to obtain complete budding and self-division, as reported in Figure 5a) [102]. In particular, we employed mixed vesicles made of POPC and oleic acid (HOA) and, as an internal chemical trigger, we used the urea–urease reaction, which is the conversion of urea in ammonia catalyzed by the Ni-protein urease [103]. The ammonia produced raises the pH (Figure 5b), leading to the deformation of the GUV: a spherical GUV elongates in a prolate form, then it becomes a pear and eventually gives two daughter vesicles connected by a narrow neck (Figure 5a). Through FRAP (fluorescence recovery after photobleaching) experiments, we achieved complete separation of the daughter vesicles, which then diffused away from each other (Figure 5c). Therefore, the enzymatic reaction induces the conversion of one spherical vesicle into two daughter vesicles that remain connected by a common membrane neck; if additional environmental triggers are applied, the neck can be broken. Interestingly, the pH increase must be accompanied by an osmotic shock (acting at the same time) to lead the vesicles to a successful budding. The higher pH inside the GUVs lumen causes oleic acid molecules to deprotonate and leave the membrane (an increase in the tension between leaflets), while the osmotic pressure deflates the vesicle (a decrease in the inner volume); the combined action of the two stimuli forces the spherical vesicles towards different equilibrium shapes until budding and/or division take place [104,105]. The influence of the surface-to-volume ratio on the shape of the GVs will be further detailed in the next section.

## 4. Modelling and Theoretical Description of Equilibrium Shapes of Giant Vesicles

The equilibrium shape of a vesicle is often determined by the minimum of the elastic energy of the membrane. The elastic energy ($W_{el}$) can be calculated as a sum of the following terms [51,106]:

(1) $$W_{el}=W_{A}+W_{r}+W_{b}+W_{G},$$

where $W_{A}$, $W_{r}$, $W_{b}$, $W_{G}$ are the *area expansion energy*, the *nonlocal bending energy*, the *local bending energy* and the *Gaussian bending energy*. In the following, we will go through each term of this equation.

The *area expansion energy* describes the stretching ability of the membrane. This energy can be approximated with a harmonic potential term [106]:

(2) $$W_{A}=\frac{\kappa }{2A_{0}}{\left(A-A_{0}\right)}^{2},$$

where κ is the area expansivity modulus, *A* is the surface and $A_{0}$ is the surface of an unstretched vesicle. During vesicle swelling—when the volume of the vesicle exceeds its volume with a non-extended sphere-shaped membrane—the expansion energy has the most relevant contribution to the elastic energy. However, in the most interesting cases, when the vesicle is flaccid—the volume of the vesicle is less than its volume with a non-compressed sphere-shaped membrane—the elastic energy is determined mostly by the bending energy terms, so the area expansion energy is often neglected [106].

The *nonlocal bending energy* occurs in multilayer systems. During the bending of a multilayer membrane, the layers will expand/compress differently relative to each other and this term takes into account this effect. According to the name, this is not a local property of the membrane; for a bilayer, the nonlocal bending energy can be calculated from the area difference of the inner and outer leaflet (ΔA) and from the preferred area difference ($\Delta A_{0}$) [50,107,108]:

(3) $$W_{r}=\frac{\kappa_{r}}{2A_{0}h^{2}}{\left(\Delta A-\Delta A_{0}\right)}^{2},$$

where $\kappa_{r}$ and *h* are the nonlocal bending constant and the distance between the neutral surfaces of the leaflets. The preferred area difference depends on the composition of the bilayer. It is worth mentioning that a small difference in the number of the particles between the layers (or in their cross-sections) can change $\Delta A_{0}$ significantly, which often manifests in significant shape changes as well. $\Delta A_{0}$ can be calculated from the cross-section of the layers ($\hat{a}$) and the number of the particles in the layer: $\Delta A_{0}=N_{outer}\cdot {\hat{a}}_{outer}-N_{inner}\cdot {\hat{a}}_{inner}$. In contrast with $\Delta A_{0}$, the area difference depends on the shape of the vesicle as well, which results in an energy contribution during shape changes.

The local and the Gaussian bending energy describes the bending energy of the membrane. The *local bending energy* can be calculated from the principal curvatures (*C*) [47,109]

(4) $$W_{b}=\frac{1}{2}\kappa_{c}\oint_{S}{\left(C_{1}+C_{2}-C_{0}\right)}^{2}\;dA,$$

where $\kappa_{c}$ is the local bending constant and $C_{0}$ is the spontaneous curvature. For a symmetric bilayer, $C_{0}$ would be zero and the layer would be flat (its radius would be infinite). With an asymmetric bilayer, the radius in a given space would be $2/C_{0}$. This is a local term because the difference between the principal curvatures and $C_{0}$ has to be integrated on the whole surface of the vesicle (*S*), and $C_{1}$, $C_{2}$, and $C_{0}$ are space dependent.

The *Gaussian bending energy* can be calculated from the principal curvatures [51,106]:

(5) $$W_{G}=\kappa_{g}\oint_{S}C_{1}C_{2}\;dA,$$

where $\kappa_{g}$ is the Gaussian bending constant. This term has to be taken into account during vesicle fusion or fission (when the number of the vesicles is changing), otherwise, its value is constant ($4\pi \kappa_{g}$); therefore, it does not change the shape of the vesicle during the energy minimisation, and it is often omitted. According to that, the elastic energy change of a flaccid vesicle during the shape determination is determined by the nonlocal and local bending energy: $W_{el}=W_{r}+W_{b}$. For simplicity, this equation is often divided with the bending energy of a sphere ($8\pi \kappa_{c}$), so we obtain the reduced elastic energy of the vesicle [57,108]

(6) $$w=\frac{1}{4}\oint_{S}{\left(c_{1}+c_{2}-c_{0}\right)}^{2}\;da+\frac{\kappa_{r}}{\kappa_{c}}{\left(\Delta a-\Delta a_{0}\right)}^{2},$$

where $c_{1}=C_{1}R_{s}$, $c_{2}=C_{2}R_{s}$, $c_{0}=C_{0}R_{s}$, the radius of the equivalent sphere is $R_{s}={\left(A_{0}/4\pi \right)}^{1/2}$, the reduced area difference and the reduced preferred area difference are $\Delta a=\Delta A/8\pi hR_{s}$, $\Delta a_{0}=\Delta A_{0}/8\pi hR_{s}$ and $da=dA/4R_{s}^{2}\pi$. There are two limiting cases according to the $\kappa_{r}/\kappa_{c}$ ratio. When this ratio is infinite, the relative area difference equals its preferred value ($\Delta a=\Delta a_{0}$) and the model is called the *strict bilayer couple* model. If this ratio equals zero, the relative area difference can differ greatly from its preferred value, and the model is called the *spontaneous curvature model*.

The equilibrium shape can be determined from the minimisation of the reduced elastic energy when the reduced area difference (Δa) and the reduced volume (ν) are constrained. The reduced area difference can be calculated from the reduced curvatures:

(7) $$\Delta a=\frac{1}{2}\oint_{S}c_{1}+c_{2}\;da,$$

and the reduced volume can be calculated from the volume of the vesicle (*V*):

(8) $$\nu =V/\left(4/3\;R_{s}^{3}\pi \right).$$

**Figure 6:**
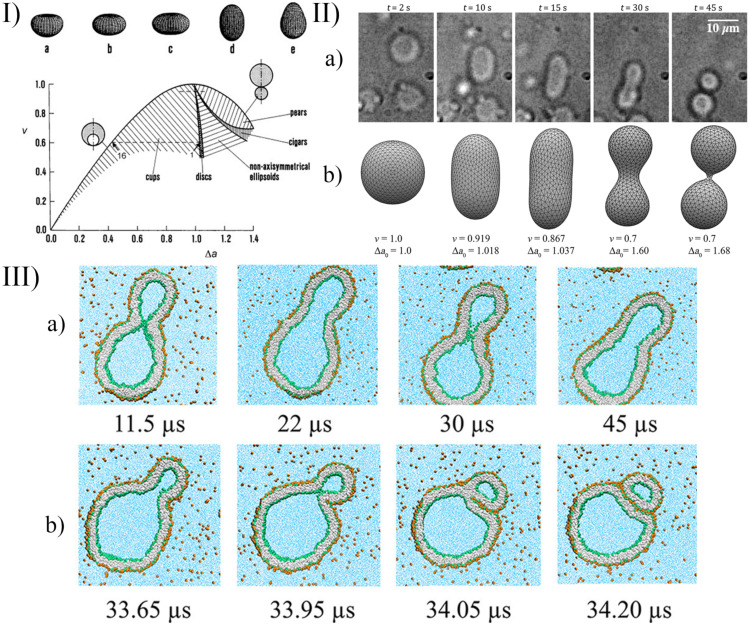
(Panel I) v-Δa phase diagram of the strict-bilayer-coupling model showing different classes of shapes; (**a**–**e**) are tridimensional representative shapes of the five classes reported in the phase diagram. Reprinted with permission from Ref. [106]. 2009, Wiley. (Panel II) Self-division of a GUV. (**a**) Shape transformation of pH-sensitive GUVs governed by the urea–urease enzymatic reaction; (**b**) numerically simulated equilibrium shapes of GUVs using the Surface Evolver software; reproduced from [104]. (Panel III) Morphological transitions of nanovesicles using coarse-grained molecular dynamics simulations; (**a**) time series of budded nanovesicle; (**b**) division of nanovesicle by fission of membrane neck. Reprinted with permission from Ref. [110]. 2021, American Chemical Society.

A few examples of the equilibrium shapes described by the strict-bilayer-coupling model are reported in the ν−Δa phase diagram in Panel I of Figure 6.

In the simulation of complex systems—when the equations can not be solved directly—the *triangularised surface approach* can be used. In this case, the surface is discretised, and it is represented by vertices connected with edges [111,112]. With this triangulated mesh, the integrals in Equation (6) can be calculated so the elastic energy of the vesicle can be computed as well. This energy is a numerical approximation so its value depends on the level of the triangulation. A denser grid gives more precise energy, but the computational cost will be higher.

The elastic energy can be minimised with a classical numerical method [111] (e.g., gradient descent, conjugate gradient, simplex method) or with *Monte Carlo* (MC) simulations [113,114]. According to the general procedure in the MC simulation, the system is perturbed slightly (the vertices are moved randomly, and the connections are rearranged), and the new state is accepted with a given probability [115]:

(9) $$p=\exp[-\left(E_{new}-E_{old}\right)/k_{B}T],$$

where $E_{new}$ and $E_{old}$ are the energies of the new and the previous state, $k_{B}$ and *T* are the Boltzmann constant and the temperature. This is a computationally expensive strategy since the possible acceptance of higher energy states slows down the convergence, but it makes the MC simulation very robust: not just the closest local energy minimum can be found (like in the gradient descent method), but farther, lower minimums become available as well. The MC simulation is a mesoscopic method, where giant vesicles can be modelled with their real size (against the smaller size available in molecular dynamics simulations), and with this method, it is possible to simulate complex systems far from their equilibrium state. For example, with MC simulation, the shape of a vesicle encapsulating a polymer [116], the interaction with a charged colloid [117], a budding due to changes in the spontaneous curvature [118], shape changes under osmotic stress [115] or the dynamic behaviour when the giant vesicles are exposed to detergents [119] can be calculated as well.

An alternative description of the dynamical behaviour of the system is possible with *Molecular Dynamics* (MD). In this case, the previous mean field description—according to the elastic energy—is not necessary, the inter- and intramolecular interactions are defined in force fields, and from the pair interactions, the force—acting on the *i*th particle—can be calculated from the gradient of the (*U*) potential: $F_{i}=\sum_{i\neq j}-\nabla U_{i,j}$. After integrating the equation of motion (e.g., with the leapfrog algorithm), the dynamical behaviour can be observed on an atomic level. The challenge with this approach is that a giant vesicle contains a huge amount of atoms, and a lot of solvent molecules have to be simulated as well. To decrease the computational cost, often coarse-grained models are used (CGMD, see Figure 6 panel III), in which some part of a molecule or even whole lipids are treated together as a single mass point [110,120]. The number of solvent molecules can be reduced with implicit solvents where the effect of the solvent molecules are included in the interaction parameters, and they are not present explicitly in the simulation box [121]. The reliability of the models decreases with these simplifications, but they are necessary—on the current computational level—to describe these huge systems. Unfortunately, even with the CGMD models, generally only small vesicles can be simulated (with ≈10 nm radius).

*Dissipative Particle Dynamics* (DPD) is an often-used method too in the simulation of vesicle shapes, fission, and fusion. This technique is based on pair interactions—similarly to MD—but it contains (next to the conservative interactions) a dissipative and a random force between the particles, which confers viscosity to the system and conditions under which flows can also be calculated [122]. These flows conform to the Navier–Stokes equations in a limiting case [123], and even the shape deformation they cause on vesicles can be estimated [124].

Although these models—based on particle–particle interactions—represent a basically different approach than the previously described elastic membrane model, the calculated shapes are in good agreement with the theoretical models and with the experiments in the case of MD [125,126,127] and DPD [125] simulations as well. The advantages of these models are that the dynamical behaviour [128] and the molecular structure [129] can be observed and some properties—which would be hard to model with other methods—e.g., the pressure and temperature effect [130] or the shape deformation caused by an osmotic shock [131], can be described. The budding and fission of nanovesicles can be studied in complex systems with both MD [110,132,133] and DPD [134,135,136] simulations as well.

As we have outlined, there are a lot of approaches to simulate the shape deformation, budding, and division. To choose between these methods, one should consider the simulation costs and the complexity level of the description, which are necessary to understand the phenomena. In the case of the particle-based methods (such as MC, MD, DPD, Brownian dynamics (BD), multi-particle collision (MPC) dynamics), the computational cost is high, but the phenomena can be modelled down to the molecular scale. With these techniques, both transient and equilibrium shapes can be studied. However, when the simulation aims only to describe the equilibrium shape of the vesicle, a molecular description is not necessary, and the double layer can be treated as an elastic membrane (such as in the spontaneous curvature model). In this case, the simulation costs are lower, and the size of the vesicle is not a limiting factor anymore. This equilibrium assumption is appropriate in many cases when the time scale of the physical or chemical stimuli, as described in Section 2 and Section 3, is large enough for the molecular level rearrangements to approach the equilibrium shape. This equilibrium shape of the vesicle can be seen in the theoretical phase diagrams (Panel I of Figure 6). If the reduced area and the reduced volume are known and the equilibrium assumption is fulfilled, the shape of the vesicle can be followed in the experimental processes using this phase diagram. These reduced parameters can be determined in dynamic simulations as a function of time as well. For example, after a chemical stimulus, the concentration inside the vesicle can be calculated based on kinetic reaction equations, and the reduced volume and reduced area can be determined from the osmotic stress and the number of lipids in the leaflets, as, for example, was achieved for pH-induced deformations [99,102,104,105]. In other words, from the initial concentrations, the equilibrium shape can be determined at any time. In less detailed simulations (when the phenomena are investigated at a bigger scale), computationally less demanding, alternative approaches can be used. For example, the basic condition and the frequency of vesicle division can even be determined based on simple geometrical considerations [137]. In such cases, a simplified model could be more effective and comprehensible since it can offer a clear insight into the key features of the phenomena.

## 5. Conclusions

**Figure 7:**
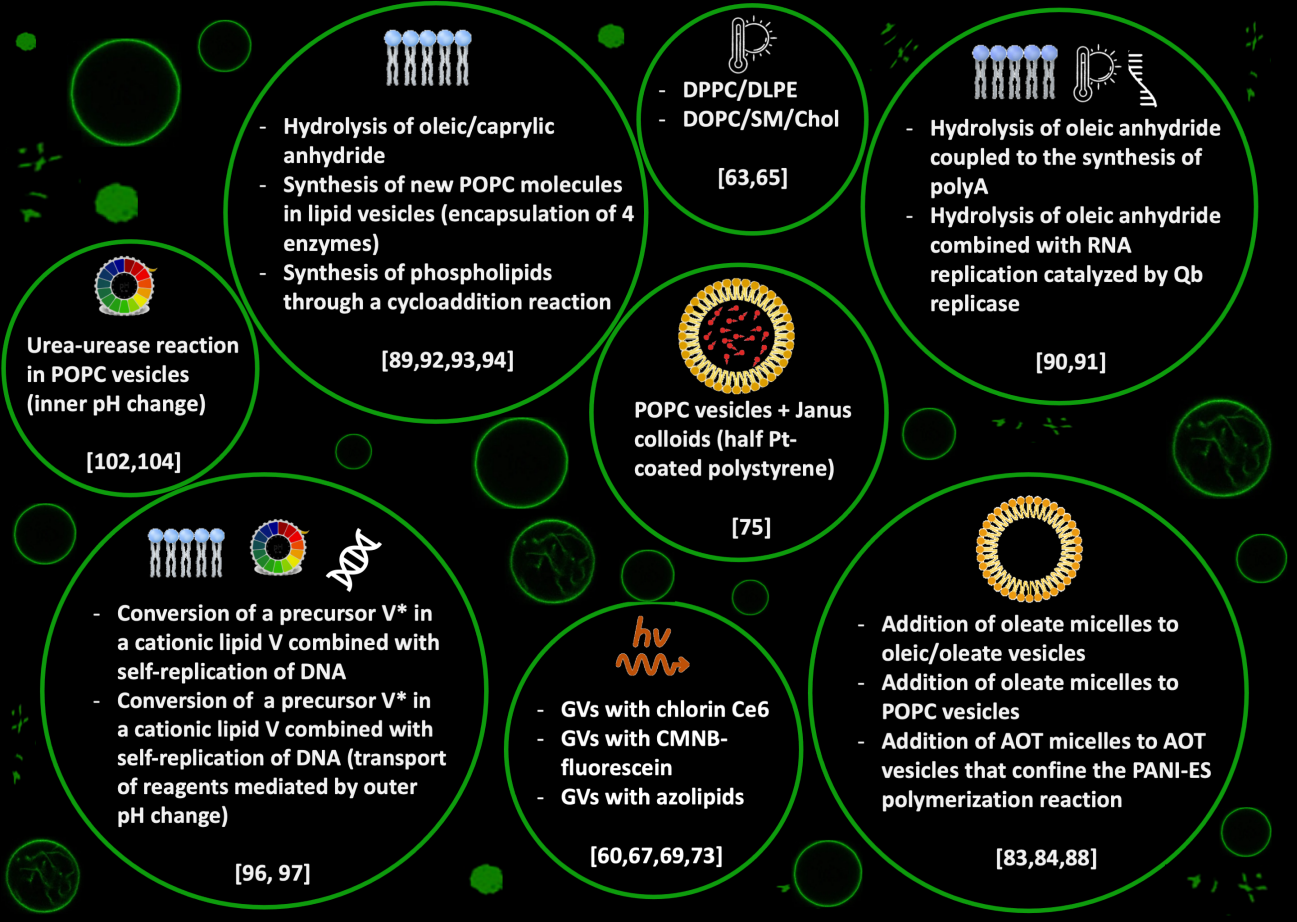
Graphical summary of the most important physical and chemical stimuli for inducing budding and self-division of GVs, as reviewed in this paper, with the corresponding principal references.

Membrane engineering is among the most studied strategies to control the behaviour of artificial cells. Depending on the properties and the composition of the membrane, in fact, it is possible to master complex functionalities of GVs, depicted here as a general model for artificial cells: in particular, tuneable permeability, shape changes, growth and division and fission. In this paper, we reviewed several studies reporting different strategies to control the equilibrium shapes—that eventually cause division—of phospholipid and/or fatty acids based GVs (a concise summary is reported in Figure 7). The integration of the experimental results (Section 2 and Section 3) with the theoretical description and the strategies for the simulation of the observed phenomena (Section 4) offer many hints for designing new stimuli-responsive membranes. At the same time, though, new questions are brought about, for example, regarding the thermodynamic and energetic landscape where these phenomena take place, an aspect that, in our opinion, has been somehow overlooked. Although GV shapes can be described as equilibrium structures, the processes that drive a GV from one shape to another need to occur far from thermodynamic equilibrium, and an input of energy and/or mass is always required. In addition to that, some of the systems reported in this review produce transient structures that are more in a steady-state rather than at the thermodynamic equilibrium; this is certainly true for light-driven deformations occurring with *azo*PCs based membranes, where, in the absence of continuous UV-light irradiation, the cis form thermally relaxes to the trans form bringing back the GV to its original state. Future research should take into consideration the thermodynamic aspects to obtain a better understanding of the division processes occurring in artificial cells.

Although relatively simple systems were considered here, which hardly involved biochemical components (except for some enzymes), the mechanisms devised allowed one to catch the innermost physico-chemical aspects of the biological processes that inspired these artificial systems. Following such a bottom-up approach, synthetic biologists and systems chemists are continuously developing new systems able to reproduce life functions, demonstrating their great value and potential for technological applications (e.g., smart materials, targeted drug delivery, biocompatible devices, etc.) and fundamental research (origin of life studies, biological mechanisms, etc.).

## Data Availability

Not applicable.
